# Evaluation of the effects of propylisopropylacetic acid (PIA) on neuronal growth cone morphology

**DOI:** 10.1016/j.neuropharm.2009.01.014

**Published:** 2009-03

**Authors:** Jakob A. Shimshoni, Emma C. Dalton, Peter Watson, Boris Yagen, Meir Bialer, Adrian J. Harwood

**Affiliations:** a Department of Pharmaceutics, School of Pharmacy, Faculty of Medicine, The Hebrew University of Jerusalem, Jerusalem, Israel; b School of Biosciences, Cardiff University, Museum Ave, Cardiff CF10 3AX, United Kingdom; c Department of Medicinal Chemistry and Natural Products, School of Pharmacy, Faculty of Medicine, The Hebrew University of Jerusalem, Jerusalem, Israel; d David R. Bloom Center for Pharmacy, School of Pharmacy, Faculty of Medicine, The Hebrew University of Jerusalem, Jerusalem, Israel

**Keywords:** Lithium, Valproate, Carbamazepine, Propylisopropylacetic acid, Neuronal growth cones, Actin cytoskeleton

## Abstract

Propylisopropylacetic acid (PIA) is a constitutional isomer of valproic acid (VPA). It has previously been found to be a weak antiepileptic, but in common with mood stabilizers, causes inositol depletion and growth cone spreading, suggesting the basis of a new series of mood stabilizers. To assess this possibility, we have compared the effects of racemic (R,S)-PIA and its individual enantiomers to those of the mood stabilizers lithium (Li^+^), VPA and carbamazepine (CBZ). Unlike Li^+^ and VPA, but in common with CBZ and (R,S)-PIA, neither (R)-PIA nor (S)-PIA enantiomer induces T-cell factor (TCF)-mediated gene expression. However, as seen for other mood stabilizers, both enantiomers are potent inducers of growth cone spreading. To investigate the mechanism for these effects, we examined changes in the actin cytoskeleton following drug treatment with Li^+^, VPA, CBZ, (R,S)-PIA or its individual enantiomers. All exhibit a re-distribution of F-actin to the growth cone periphery, a feature of spread growth cones. (R,S)-PIA has the strongest effect as it also elevates F-actin polymerization at the cell periphery. This change in the actin cytoskeleton is associated with a substantial increase in F-actin-rich protrusions on the surface of the growth cone and in its close vicinity. These results demonstrate an effect of (R,S)-PIA on the neuronal actin cytoskeleton shared in common with other mood stabilizers, and suggest a potential to induce structural changes within the CNS.

## 1 Introduction

Despite an intense research effort, the development of more potent and effective mood stabilizers has been slow. A major barrier to progress is our lack of molecular understanding of the mechanisms that underlie both the origin and the treatment of bipolar disorder. As no available models can replicate both aspects of mania and depression and therefore fail to mimic the hallmark of the illness, alternative approaches are being explored for identifying potential new mood stabilizers (Cryan and Slattery, 2007; Machado-Vieira et al., 2004).

Williams et al. (2002) have developed a cellular model based on the regeneration of sensory neurons (Williams et al., 2002). Here, cultured explants of rat dorsal root ganglions (DRGs) are allowed to regenerate axons. The growth cones at the termini of these regenerating axons are sensitive to drug treatment, and the mood stabilizers lithium (Li^+^), valproic acid (VPA) or carbamazepine (CBZ) induce an increase in growth cone spreading. This change in morphology can be measured by calculation of growth cone spread areas. Using this as a bioassay, we recently found that PIA, a constitutional isomer of VPA, also induces growth cone spreading (Shimshoni et al., 2007a). This raises the question of the mechanism of this and other mood stabilizers on growth cone behaviour.

Both Li^+^ and VPA mimic the effects of Wnt signaling by inducing T-cell factor (TCF)-mediated gene expression (Gould et al., 2007; Lucas et al., 1998; Phiel et al., 2001). As Wnt signaling is crucial for the development of the CNS and is directly involved in neurogenesis and neuronal differentiation, it remains a potential target of mood stabilizers (Lucas et al., 1998). Li^+^ stimulates Wnt signaling via inhibition the downstream protein kinase glycogen synthase kinase-3 (GSK-3) leading to increased β-catenin protein concentrations (Harwood and Agam, 2003; Klein and Melton, 1996; Ryves and Harwood, 2001). Elevated β-catenin binds to and activates the transcription factor TCF to control gene expression. In some, but not all cellular systems, VPA can indirectly increase β-catenin expression via inhibition of histone deacetylase (HDAC) (Phiel et al., 2001; Ryves et al., 2005), again stimulating TCF-mediated gene expression.

There are a number of problems with this hypothesis. First, GSK-3 mediates changes in microtubule dynamics through phosphorylation of microtubule associated proteins, such as MAP1B and *tau* (Lovestone et al., 1999; Owen and Gordon-Weeks, 2003; Sang et al., 2001). However, whilst Li^+^ treatment or Wnt stimulation causes growth cone spreading with an associated change in microtubule morphology, this is not the case for the alternative mood stabilizers VPA and CBZ (Williams et al., 2002). These drugs cause the same degree of growth cone spreading, but no major change of the microtubule cytoskeleton. Second, previous results have shown that CBZ does not increase β-catenin expression, but is a potent inducer of growth cone spreading (Ryves et al., 2005; Williams et al., 2002). Third, (R,S)-PIA induces growth cone spreading, but has no effect on TCF-mediated gene expression (Shimshoni et al., 2007a). These results argue against a common mechanism for all mood stabilizers on either Wnt signaling or regulation of microtubule dynamics within the neuronal growth cone.

In contrast, the effects of all drugs, Li^+^, VPA, CBZ and (R,S)-PIA, can be reversed by addition of myo-inositol, suggesting a mechanism via inositol depletion. This mechanism was first suggested by Berridge et al. (1989) to explain the effects of lithium on inositol-1,4,5-triphosphate (InsP_3_) mediated signaling. Li^+^ is a non-competitive inhibitor of inositol-monophosphatase (IMPase) (Atack et al., 1995; Hallcher and Sherman, 1980) and leads to a decrease in the cellular concentration of myo-inositol, which can be reversed by inositol uptake (Mustelin et al., 1986). More recently VPA has been shown to deplete cellular inositol via inhibition of inositol synthase (Shaltiel et al., 2004; Silverstone et al., 2002), and CBZ to cause inositol depletion (Williams et al., 2002) by an unknown mechanism. Finally, our recent studies indicate that (R,S)-PIA also reduces the cellular concentrations of (InsP_3_) (Shimshoni et al., 2007a). This suggests that mood stabilizers may act via a common mechanism of suppression of inositol phosphate based signaling, however how it affects growth cone morphology is currently unknown.

We show here that the PIA enantiomers (S)-PIA and (R)-PIA induce both growth cone spreading and actin polymerization, but have no effect on TCF-mediated gene expression. We also report the characterization of the effects of (R,S)-PIA and the mood stabilizers, Li^+^, VPA and CBZ on the growth cone cytoskeleton. We observe a common effect of all drugs on actin cytoskeleton and an induction of actin-rich protrusions associated with similar changes in growth cone morphology.

## 2 Methods

### 2.1 Materials

Solvents and drugs were purchased from Sigma–Aldrich (UK). (R,S)-propylisopropylacetic acid (PIA) and its two enantiomers (S)-PIA and (R)-PIA were synthesized according to the synthetic procedures previously described in Shimshoni et al. (2007b). All compounds used in the *in vitro* experiments were dissolved in water or ethanol to result in stock solutions of 0.2 M. Texas-Red^®^-X phalloidin for F-actin staining was purchased from Invitrogen, and monoclonal anti α-tubulin antibody conjugated to FITC was obtained from Sigma–Aldrich (UK).

### 2.2 Dorsal root ganglion explant culture

Dorsal root ganglia (DRG) were dissected from the spinal cord of 1 day old Sprague–Dawley rats and cultured individually on poly-ornithine/laminin coated coverslips in serum-free medium at 37 °C with 5% CO_2_ (Bottenstein et al., 1979). Media were supplemented with mouse NGF-7s (25 ng/ml; the optimal concentration for growth stimulation was determined for each batch) in order to promote neuron survival and axon outgrowth (Williams et al., 2002). After attachment for 24 h, the antimitotic agent cytosine β-arabinofuranoside (Ara-C; 10 μM) was added for 24 h to kill non-neuronal cells. DRG explants were then changed to fresh serum-free media for a further 24 h. Drugs were added at 2 mM NaCl (control), 2 mM LiCl, 3 mM VPA, 85 μM CBZ, 1 mM (R,S)-PIA or one of its two enantiomers (S)-PIA (1 mM) and (R)-PIA (1 mM). Mood stabilizer concentrations are 1.5 times the therapeutic level. Time lapse video-microscopy was carried out using an IX70 inverted DIC microscope (Olympus) at 20× magnification and recorded using an Orca ER CCD camera (Hamamatsu) and SimplePCI v6.0 image software (Digitalpixal).

### 2.3 Immunocytochemistry

DRGs were washed with PBS and fixed for 10 min in 4% formaldehyde. Samples were then washed with PBS followed by 0.1% sodium borohydride and permeabilized in PBS solution containing 0.2% Triton X-100 and 1% BSA. Samples were washed with PBS and blocked for 20 min in 1% BSA in PBS. Sequential staining was carried out with a 1:400 dilution of anti-α-tubulin monoclonal antibody conjugated to FITC for 1 h at room temperature. Samples were washed three times followed by a 1:40 dilution of Texas-Red^®^-X phalloidin for 20 min at room temperature. Samples were washed with PBS and mounted with 35 μl of a mounting medium containing antifade reagent (Vectashield^®^ Hard Set). Samples were imaged at 63 × 1.6 in oil with simultaneous excitation at 494 nm and 596 nm, and measurement at 516 nm for FITC and 620 nm for Texas-Red^®^. Images were analyzed using SimplePCI v6.0 image software program and data collected using a double blind protocol. Between 10 and 50 growth cones were formed per DRG. Each experiment comprised 1–2 DRG and was repeated in triplicate; giving between 80 and 120 growth cone measurements per drug.

### 2.4 TCF promoter activity

Activation of TCF promoter activity was monitored using the TOPflash assay (Korinek et al., 1997). HEK293 epithelial cells transfected with TCF-luciferase reporter plasmid were seeded at 1 × 10^4^ cells per well of 96 well black-walled dishes in 50 μl DMEM medium and incubated at 37 °C for 48 h. Drugs were added on the third day and cells incubated for a further 48 h. Cells were lysed in GLO lysis buffer (Promega, Southampton, UK) and luciferase substrate (BrightGLO, Promega, Southampton, UK) was added. Luminescence measurements were made on a Fluostar multi-platform plate reader (BMG Labtech, Aylesbury, UK).

### 2.5 Statistical analysis

Analysis of growth cone morphometrics was made using a Kruskal–Wallis analysis of variance test followed by Dunn's multiple comparison test for post hoc pairwise comparison with the control value. Analysis of F-actin fluorescence was made using a parametric one-way ANOVA followed by a conservative Bonferroni Multiple Comparisons test. All statistical analyses were performed with GraphPad InStat, version 3.01 (GraphPad Software, San Diego, CA, USA). Data are expressed as mean ± S.D. A *p* value of ≤0.05 was considered significant.

## 3 Results

### 3.1 (R,S)-PIA isomers do not induce TCF-mediated gene expression

**Fig. 1:**
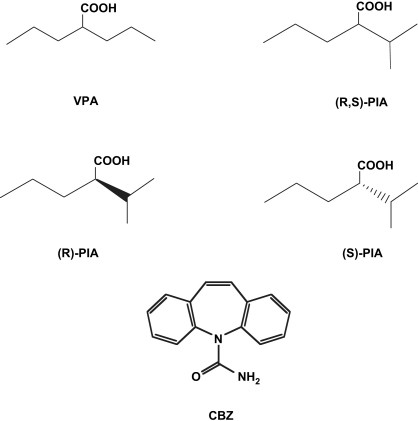
Chemical structures of compounds used in this study. VPA, valproic acid; (R,S)-propylisopropylacetic acid, (R,S)-PIA; (R)-propylisopropylacetic acid, (R)-PIA; (S)-propylisopropylacetic acid, (S)-PIA; carbamazepine, CBZ.

**Fig. 2:**
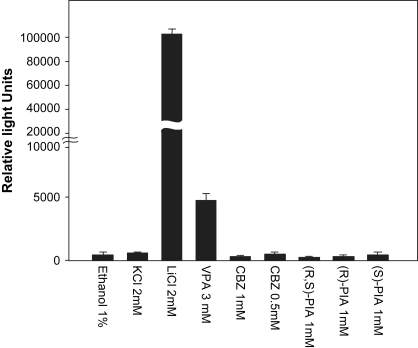
Induction of TCF-mediated gene expression. Cells were grown for 48 h in complete medium in the presence of lithium (2 mM), CBZ (1 and 0.5 mM), VPA (3 mM), (R,S)-PIA (1 mM) or one of its two enantiomers, (R)-PIA (1 mM) and (S)-PIA (1 mM). The histograms show the relative light unites for the corresponding drugs assayed in triplicates (Mean ± SD).

Previous analysis indicated that unlike Li^+^ and VPA, (R,S)-PIA had no effect on TCF-mediated signaling, and hence is not an inducer of Wnt signaling. As a chiral molecule, (R,S)-PIA exists as two enantiomers, (R)- and (S)-PIA (Fig. 1). Thus it is possible that enantioselective activity was masked in these earlier experiments using racemic-PIA. As the relationship between mood stabilizers and Wnt signaling is contentious, it was important to eliminate any possibility that (R,S)-PIA could induce TCF-mediated gene expression via one of its two enantiomers. We therefore analyzed whether either (R)- or (S)-PIA behaved differently in a TCF-luciferase-based reporter assay in HEK293 cells (the TOPflash assay). As previously observed, Li^+^ induced a large (10^5^-fold) increase in TCF-luciferase expression, whereas VPA produced a smaller 5000-fold increase (Fig. 2). In contrast neither CBZ, (R,S)-PIA nor the PIA enantiomers induced an increase in TCF-luciferase expression. These results confirm that no form of PIA can induce TCF-mediated expression.

### 3.2 (R,S)-PIA and its two enantiomers increase growth cone spreading

**Fig. 3:**
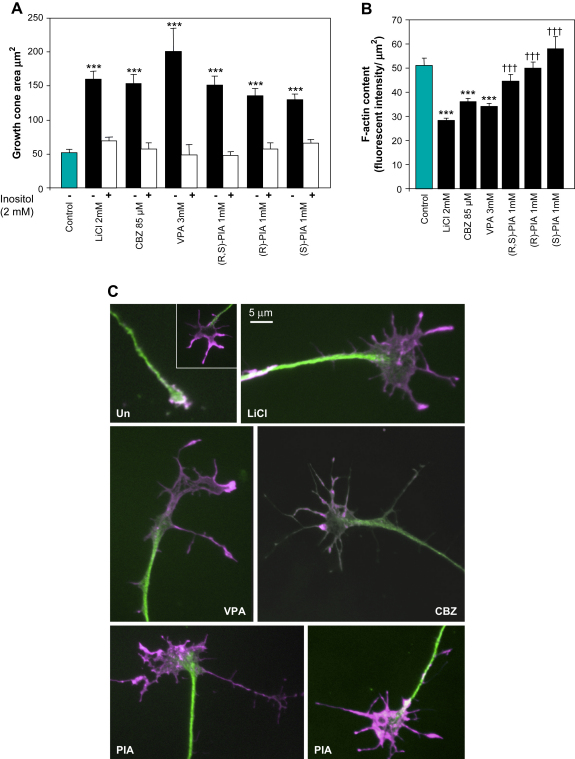
The effects of lithium, VPA, CBZ, (R,S)-PIA and its individual enantiomers (R)-PIA and (S)-PIA on growth cone morphology. (A) Histogram of the growth cone area (μm^2^) (Mean ± SD). Grey bar, untreated control; white bar, drug treatment plus 2 mM myo-inositol. (B) Histogram of the F-actin content within growth cone, calculated as fluorescence intensity of growth cones stained with Texas-Red^®^ phalloidin normalized to growth cone area (Mean ± SD). (C) Representative images of growth cones double-stained with Texas-Red^®^ phalloidin against F-actin (magenta) and with FITC-conjugated anti-α-tubulin antibody against microtubule (green). Un: untreated controls, a contracted growth cone is shown in the main image, a spread growth cone is shown in insert; LiCl: 2 mM LiCl; VPA: 3 mM VPA; CBZ: 85 mM CBZ; PIA: 1 mM (R,S)-PIA. Bar = 5 μm. Sample groups comprise: untreated control, 76 growth cones derived from 4 DRG explants; LiCl, 118 growth cones from 5 DRG explants; CBZ, 97 growth cones from 4 DRG explants; VPA, 90 growth cones from 4 DRG explants; (R,S)-PIA, 81 growth cones from 4 DRG explants; (R)-PIA, 100 growth cones from 4 DRG explants; (S)-PIA, 81 growth cones derived from 4 DRG explants. Significance tests: ***p < 0.001 for experiment vs. untreated control; †††*p* < 0.001 PIA species experiment vs. Li^+^.

Previously Li^+^, VPA, CBZ and PIA have been shown to cause spreading of growth cones of cultured DRG neurons (Shimshoni et al., 2007a; Williams et al., 2002). To further examine the structure–function relationship between VPA and (R,S)-PIA, we examined growth cone behaviour following treatment with (S)- and (R)-PIA. Growth cones were treated with a range of drugs and scored by measuring the spread area of each growth cone. A significant increase in growth cone area was observed following treatment with 2 mM LiCl, 3 mM VPA and 85 μM CBZ. However, no significant increase in growth cone spreading was observed when these concentrations were reduced. The constitutional isomer (R,S)-PIA and its two individual enantiomers, (R)- and (S)-PIA strongly increased growth cone spreading down to concentration of 1 mM, significantly lower than VPA, suggesting a higher potency of this VPA isomer than VPA itself (Fig. 3A). Again, we could observe no difference in potency or effect between racemic-PIA and its individual enantiomers. In all cases, addition of 2 mM myo-inositol reversed the drug effects, consistent with a mechanism via inositol depletion (Fig. 3A).

To further compare the effects of each drug, we used time lapse video-microscopy to investigate growth cone dynamics of treated and untreated cultures (Supplementary movie). The majority of growth cones in untreated control cultures were small and contracted. They showed cycles of membrane expansion and contraction, moving forward as long projections that arise from the relatively more spread phases of growth cone morphology. However to make a fair comparison with the larger spread growth cones of drug-treated cultures, we also recorded the less frequent spread growth cones present within the untreated culture. Interestingly, these growth cones remained relatively large throughout their movement cycle and at no point did they contract to the size of the more frequent smaller growth cones. This indicates that large and small growth cones do not interconvert as the growth cone moves forward. The larger growth cones were not static, instead they showed a great deal of activity, extending and contracting both large membranous lamellipodia and thinner filopodial-like projections. We also noted that these larger growth cones moved forward at a slower rate than the smaller growth cone type. For each drug treatment, Li^+^, VPA, CBZ and PIA, the growth cones showed the same behaviour exhibited by the larger untreated growth cones. This indicated that all drugs had the same basic effect on growth cones, increasing the frequency of enlarged, spread morphologies rather than causing a dramatic change in growth cone dynamics.

### 3.3 Mood stabilizers alter the F-actin distribution within the growth cone

Growth cone morphology is determined by dynamic changes of F-actin and microtubule based cytoskeleton (Dent and Gertler, 2003). Previous studies have concentrated on the effects of mood stabilizers on microtubule dynamics. Li^+^ increases microtubule stability, causing characteristic changes in microtubule morphology (Goold et al., 1999). VPA and CBZ however do not induce an equivalent change in microtubule morphology, instead the microtubules show a splayed morphology where they leave the end of the axon, and stain diffusely within the centre of the grow cone (Williams et al., 2002). Given the lack of a common effect on microtubule dynamics, we focused on changes in F-actin distribution.

Initially, we examined global changes in F-actin content through intensity of phalloidin staining. This was done by quantification of fluorescence intensity of individual growth cones stained with Texas-Red^®^-X phalloidin (collected in the linear range) divided by their spread area. This normalizes for the different size distribution of treated and non-treated neurons. Li^+^, VPA and CBZ had approximately 60–70% of the F-actin content per unit area than non-treated controls (Fig. 3B). In contrast, the F-actin content of growth cones treated with 1 mM (R,S)-PIA (data not shown) or its two enantiomers (Fig. 3B) was significantly higher than those treated with the other drugs. In fact there was no significant difference from the untreated control (Fig. 3B).

To understand these differences, we examined the spatial distribution of F-actin and microtubules within growth cones (Fig. 3C). In the majority of untreated cells F-actin forms a predominant part of the growth cone, with rest occupied by microtubules. However, as the untreated cells have a contracted morphology rather than the spread morphology of those treated with drugs, we also examined the small number of untreated growth cones that had a large spread area. In contrast to contracted growth cones, these showed a core region enriched in neither F-actin nor microtubules, this central domain of growth cones is enriched in cytoplasm, mitochondria and accumulates membrane to facilitate extension of the axon. A similar distribution is seen in drug-treated neurons. In these spread growth cones microtubules were concentrated in the axon, whereas F-actin was enriched at the periphery of the growth cone, and only present at a low level within the central region of the growth cone. This non-heterogeneous distribution of F-actin within the growth cone caused the reduction in F-actin polymerization when measured as total fluorescence per unit area as the large majority of growth cones treated with Li^+^, VPA or CBZ had the peripheral F-actin distribution, consistent with their larger size (Fig. 3C). This explains why these drugs caused a decrease in average F-actin content per unit area than untreated controls.

Examination of (R,S)-PIA (Fig. 3C), (R)-PIA or (S)-PIA (data not shown) treated cells again showed the re-distribution of F-actin observed with the other drugs and consistent with the spreading effect of this drug, however the fluorescence intensity within the periphery appeared stronger. This can explain why PIA treatment had a higher overall fluorescence compared to other mood stabilizers and showed no significant reduction in overall growth cone fluorescence compared to untreated controls; i.e. the reduction of fluorescence in the central region of the (R,S)-PIA treated growth cones is masked by elevated fluorescence at the periphery. Consistent with this we found that neurons treated with (R,S)-PIA or its enantiomers showed significantly more fluorescence than those treated with Li^+^, VPA or CBZ (Fig. 3B). We therefore conclude that induction of growth cones spreading following drug treatment is coupled to alteration in actin dynamics. This effect is strongest with (R,S)-PIA and its enantiomers, which are the most potent drugs at inducing growth cone spreading.

### 3.4 Induction of F-actin-rich protrusions on the growth cone

The most striking feature of drug treatment on growth cone morphology was the induction of F-actin-rich protrusions that extend from both the growth cone and the axon in the vicinity (within 65 μm) of the growth cone. These protrusions stain strongly with Texas-Red^®^-X phalloidin, but only weakly with α-tubulin. There is a small degree of co-localization of F-actin and α-tubulin at the distal ends of the protrusions, however as there is no enrichment of α-tubulin compared to the central region of the growth cone, we conclude that these structures arise through changes in the actin cytoskeleton, rather than local generation of microtubules. The protrusions observed from the proximal region of the axon appeared to be more mixed with the majority of protrusions enriched in F-actin, but a smaller proportion enriched in α-tubulin.

**Fig. 4:**
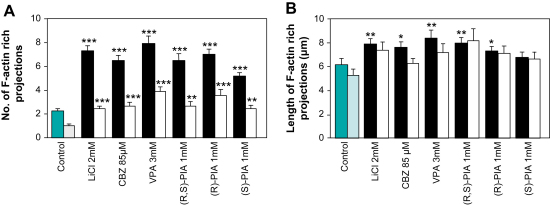
The effects of lithium, VPA, CBZ, (R,S)-PIA and its two individual enantiomers (R)-PIA and (S)-PIA on actin-rich protrusions. (A) Histogram to show the mean (±SD) number of F-actin-rich protrusions per growth cone (dark bars) or along an axonal section close to (<65 μm) the growth cone (light bar). (B) Histogram to show mean length of the protrusions extending from the growth cone or an axonal section close to (<65 μm) the growth cone (light bar). Sample groups comprise: untreated controls, 76 growth cones from 4 DRG explants; 2 mM LiCl, 118 growth cones from 5 DRG explants; 85 μM CBZ, 97 growth cones from 4 DRG explants; 3 mM VPA, 90 growth cones from 4 DRG explants; 1 mM (R,S)-PIA, 81 growth cones from 4 DRG explants; 1 mM (R)-PIA, 100 growth cones from 4 DRG explants; 1 mM (S)-PIA, 81 growth cones from 4 DRG explants. Total data were analyzed by a Kruskal–Wallis test, followed by Dunn's multiple comparison test of each experiment vs. control: *p < 0.05; **p < 0.01; ***p < 0.001.

Growth cones of drug-treated neurons possessed an average of between 5 and 8 protrusions with as many as 19 present on a single growth cone, whilst untreated neurons possessed an average of 2 with no more than 6 present on a single growth cone (Table 1; Fig. 4A). The increase in protrusions could arise either through increased initiation of their formation or altered dynamics of their growth. We observed only a small increase in protrusion length on the growth cone and no increase on those on the axon (Fig. 4B). This argues that although there may be some change in their growth dynamics, the major effect of drug treatment is to increase the rate of initiation of these F-actin structures.

## 4 Discussion

The identity of the cellular target of mood stabilizers remains disputed. One argument proposes that the Wnt signaling pathway is the important mood stabilizer target. This is supported by the observations that Li^+^ inhibits GSK-3 kinase activity and causes neuronal growth cone spreading with an associated change in microtubule dynamics (Goold et al., 1999; Klein and Melton, 1996; Ryves and Harwood, 2001). There is no doubt that GSK-3 is neurologically active, as GSK-3 hypomorphic mutations and GSK-3 inhibitors cause behavioural abnormalities in mice that are similar to those induced by treatment with antidepressant drugs (Beaulieu et al., 2004; Gould et al., 2004; Kaidanovich-Beilin et al., 2004; O'Brien et al., 2004). GSK-3 mediated signaling is also involved in mechanisms of neuroprotection and has been associated with schizophrenia (Lovestone et al., 2007; Toledo et al., 2008). However, there is currently no direct evidence to link GSK-3 to bipolar mood disorder. On the other hand, the less commonly used mood stabilizer CBZ also causes growth cone spreading, but with no change in microtubule structure or inhibition of GSK-3. CBZ has previously been shown to have no effect on β-catenin expression (Ryves et al., 2005), and we show here that CBZ does not induce TCF-mediated gene expression, eliminating any possibility of a β-catenin independent mechanism of stimulating Wnt signaling.

The situation with VPA is less clear. Initial reports suggested that VPA may directly inhibit GSK-3 (Chen et al., 1999), however a number of further investigations have demonstrated this not to be the case (Eickholt et al., 2005; Phiel et al., 2001; Ryves et al., 2005). VPA has been shown to alter gene expression via inhibition of histone deacetyalses (HDACs), and, in some but not all, cell contexts VPA can induce expression of β-catenin leading to stimulation of TCF-mediated gene expression (Phiel et al., 2001; Ryves et al., 2005). Previous reports, and those reported here, show that the effect of VPA on growth cone morphology is like that of CBZ (Williams et al., 2002) and, with the exception of one report (Hall et al., 2002), VPA and CBZ were shown to cause growth cone spreading without a change in microtubule dynamics. The VPA isomer (R,S)-PIA again has no effect on HDAC activity and only weak antiepileptic activity, but is a potent inducer of growth cone spreading (Shimshoni et al., 2007a). We can now exclude the possibility that an enantiomer specific effect on TCF-mediated gene expression was missed in this earlier experiment, and can conclude without doubt that the VPA constitutional isomer (R,S)-PIA can induce growth cone spreading without activation of Wnt signaling. It remains to be seen how (R,S)-PIA performs in animal models of bipolar disorder or patients.

To understand how the mood stabilizers, Li^+^, VPA and CBZ, and (R,S)-PIA cause changes in growth cone morphology, we examined underlying changes in the actin cytoskeleton. We found two effects common to all drugs. First, we found that growth cone spreading is associated with localization of F-actin to the periphery of the cone. This is a feature of all spread growth cones, including those of the few observed in untreated control. Both qualitative examination of images and quantitative measurements of the intensity of phalloidin staining indicated that (R,S)-PIA induces elevated levels of F-actin. As (R,S)-PIA exhibits its effects at a lower concentration than VPA and therefore is the most potent inducer of growth cone spreading, these results suggest that drug treatment can drive local F-actin polymerization leading to changes in growth cone morphology.

Second, we find that all drugs induce a substantial increase in F-actin enriched protrusions that extend from the growth cone or its close vicinity. They are present at a low frequency on untreated DRG neurons, and as there is only a marginal increase in length, but a large increase in number following drug treatment, we conclude that mood stabilizers induce formation of these protrusions rather than have a major effect on the stability of those already extant. The morphology, restricted distribution and lack of α-tubulin enrichment indicate that these structures are not the same as the interstitial branching previously observed following Li^+^, but not VPA and CBZ treatment (Williams et al., 2002). As they are longer than 2 μm and enriched in F-actin, they resemble filopodia located on the axonal growth cone, which forms at the leading edge of motile cells or during early stages of synaptogenesis (Waites et al., 2005). However, unlike reports for filopodia, they can branch and in places become thickened. As a consequence the actual identity of these protrusions is unclear. Their presence does however indicate that (R,S)-PIA and mood stabilizers have a substantial effect on the neuronal actin-based cytoskeleton.

As enlarged growth cones are observed on presynaptic terminals during synaptogenesis, axon bifurcation and during periods of sensing guidance cues from the environment (Eickholt et al., 2004; Williams et al., 2004), these findings support the hypothesis that mood stabilizers modify the neural architecture. Such changes could lead to increased local neuronal interactions and connectivity, thereby reversing the reduced neuronal density observed in bipolar patients (Adler et al., 2005). This could occur via the longer-term events of neuronal morphogenesis. Alternatively functional changes could have more immediate effects on synapse function of the presynaptic terminal and structural plasticity (Luo, 2002).

Although the actin cytoskeleton is not essential for axonal elongation, it is an important component of growth cone guidance. One possibility is that mood stabilizers alter responses to extracellular signals leading to a change in the network of neuronal interactions. This re-wiring could occur during periods of neuronal development, including the establishment of new neuronal interactions following neurogenesis. Indeed several studies have demonstrated a reversal of the reduction in grey matter volume in the prefrontal cortex and an increased neuronal and glial density, following treatment with mood stabilizers (Frey et al., 2004, 2007). On a faster timescale, regulation of actin polymerization is also important for synapse formation of pre- and post-synaptic terminals, controlling both remodeling of the post-synaptic membrane and vesicle transport and the movement of dendritic spines, which form the majority of the post-synaptic sites within the mammalian brain. It is possible that drug-mediated changes in actin dynamics may alter the processes governing synapse formation of pre- and post-synaptic terminals, stability or function. Finally, as filopod formation is an early stage of synaptogenesis of presynaptic terminals, the increase in actin-rich protrusions observed in drug-treated growth cones may reflect a switch from axonal growth to synapse formation.

In conclusion, this study using (R,S)-PIA, its individual enantiomers and mood stabilizers further indicates a Wnt independent change in neuronal behaviour. In contrast to earlier work that focused on the effects of Li^+^ on microtubule dynamics, this current study argues for a common effect for mood stabilizers on the actin cytoskeleton, which may lead to changes in neuronal morphology and plasticity in the CNS. (R,S)-PIA and its individual enantiomers are potent inducers of this effect, and suggest their potential as new mood stabilizers.

## Figures and Tables

**Table 1:** The number of actin-rich protrusions per growth cone.

	Range	Mean number
Control	0–6	2
3 mM LiCl	2–16	7
2 mM VPA	2–19	8
85 μM CBZ	2–15	6
1 mM (R,S)-PIA	0–17	6
1 mM (R)-PIA	4–17	7
1 mM (S)-PIA	1–11	5

## Appendix Supplementary information

A composite time lapse movie (Quicktime) of typical growth cones recorded at 1 frame per 30 s for up to 15 min.
